# Increased Mortality and Morbidity Due to the Increase in Border Wall Height

**DOI:** 10.7759/cureus.51113

**Published:** 2023-12-26

**Authors:** Sharmeen Azad, Andrew McCague, Austin Henken-Siefken

**Affiliations:** 1 Surgery, Desert Regional Medical Center, Palm Springs, USA; 2 Surgery, Western University of Health Sciences, Lebanon, USA; 3 Trauma and Acute Care Surgery, Desert Regional Medical Center, Palm Springs, USA

**Keywords:** mexican border wall, fatalities, lower extremity injuries, trauma, border wall height, border wall fall

## Abstract

Objective

Our retrospective cohort study focuses on the differences in the severity of injuries sustained from border wall falls before and after wall height increase. Severity of injuries is categorized by injury severity score (ISS), length of stay in the hospital (LOS), ventilation, intensive care unit (ICU), and surgery. The purpose of this study is to underline the medical consequences of extending the US-Mexico border wall. Specifically, we focused on the severity of injuries that are seen in trauma centers near the US-Mexico border. We propose that the rise in trauma cases from the border wall is associated with the extension of the border wall.

Methods

This IRB-approved, retrospective cohort study included all patients that were admitted to Desert Regional Medical Center, a level 1 trauma center in Palm Springs, California, United States. Patients were admitted between March 2016 and December 2021, after sustaining a fall from the border wall. The fall of the height ranged from 15 to 30 feet. Patients were assigned to pre-2020 or post-2020 subgroups, based on time of admission. The total number of admissions, ISS, LOS, surgeries, ventilation, and ICU services were compared.

Results

Injuries from border wall falls grew 1250% from 2016 to 2021 (4 vs 50 admissions). When comparing the two subgroups, hospital admissions (20 vs 84) and ISS (9 vs 15) have also risen dramatically. Of all the variables compared, the days spent in the ICU proved to be statistically significant at a p-value of 0.02. Although the remaining data was not statistically significant, there still remains a trend of increasing injuries that are also more severe in presentation, requiring more interventions.

Conclusions

The increase in border height has led to a record-high number of admissions and severity of injuries. This study shows that increasing the border wall height has led to a public health crisis and underlines the profound impact that political decisions have in the medical field.

## Introduction

Migrants have risked their lives for many years to cross the US-Mexico border in the hopes of starting a new life. The establishment of the US-Mexico border was initiated in 1994, with the purpose of decreasing illegal immigration [1]. This initial border wall established under the Clinton administration showed that the United States was serious about getting illegal immigration under control. As such, the danger of crossing the border has only grown with time. With the signing of the Border Security and Immigration Enforcement Improvements executive order during the Trump administration, the 406-mile border wall that was 6-17 feet tall was replaced with one that is 30 feet tall [2]. These changes have made falls more dangerous and led to injuries that have cost hospitals a significant amount of money [3]. These projects aimed at heightening the border wall began in June 2018 and concluded in December 2019. As a result, the existing wall, previously only 10 feet tall, was increased to a height of 18 feet, and the new secondary wall was constructed to a height of 30 feet tall [2].

Previous research has indicated that higher falls may result in more severe injuries and higher mortality rates, raising concerns about the impact of a higher border wall on traumatic injuries brought on by border crossing attempts [2]. However, there is currently no empirical data from population-wide person-level data available to evaluate the association between health outcomes and the construction of border walls [4]. By comprehending the impact of border wall falls prior to and following the height increase, we can more clearly understand the detrimental consequences this will have on migrants who continue to attempt to cross the border and seek asylum. It is important to note that despite efforts to stop immigration, border crossings are still rising [2].

Our retrospective study included 108 patients that were admitted for border wall falls to Desert Regional Medical Center, a level 1 trauma center, between March 2016 and December 2021. These patients were de-identified, and data on their admission was collected. We aimed to focus on the differences in the severity of injuries sustained from border wall falls before and after wall height increase. Severity of injuries is categorized by injury severity score (ISS), length of stay in the hospital (LOS), ventilation, intensive care unit (ICU), and surgery. The purpose of this study is to underline some of the medical consequences of extending the US-Mexico border wall. Specifically, we focused on the severity of injuries that are seen in Desert Regional Medical Center, which is about 150 miles north of the US-Mexico border [5,6]. We propose that the rise in trauma cases from the border wall is associated with the extension of the border wall.

This study can help underline the significant influence political actions have on the medical community. Certain political actions have tremendous societal consequences, and this is a prime example. Increasing the border wall height has led to a humanitarian crisis that is felt by many medical professionals in the trauma centers near the border.

## Materials and methods

This retrospective cohort study was approved by the MetroWest Medical Center Institutional Review Board (approval number: 2020-111). The study included all patients that were admitted after a fall from the US-Mexican border wall to Desert Regional Medical Center, a level 1 trauma center in Palm Springs, California, United States, between March 2016 and December 2021. De-identified data collected on the patients from the trauma registry included age, sex, ISS, LOS, surgery status, ventilation status, and ICU admission.

We defined the pre-wall and post-wall time frame as beginning of 2020, as December 2019 was when the border wall height had been fully extended. The total group of patients was separated by admission date with 22 that were admitted before January 2020 and 86 admitted after January 2020. Data was collected on ISS from the de-identified database. Statistical analysis was complete with Microsoft Excel™ (Microsoft Corporation, Redmond, Washington, United States). All tests were two-sided with ɑ=0.05. The continuous variables were compared using the independent samples t-test. We analyzed the ISS in patients admitted before 2020 and compared them to the patients admitted after 2020. We also assessed LOS, days spent in ICU, and days on ventilation between the two groups and found a p-value. To assess the statistical significance to determine how many patients required surgery, we gave a numerical value of "0" to those who did not receive the service and "1" for those who received the service. We did all of the calculations from the data using Microsoft Excel™.

## Results

We present the demographics of the patients, as shown in Table 1. Of the 108 admissions from the border wall, 22 were in the pre-height extension period (pre 2020) and 86 in the post-height extension period (post 2020). The mean age was similar between both groups (33.2 vs 30.12 years; p=0.16). There were a total of 75 males and 33 females. In the pre-2020 group, there were 54.5% male vs 45.4% female, and in the post-2020 group, there was 77.4% male vs 25.6% females.

**Table 1:** Demographics SD: standard deviation

Factor	All	Pre 2020	Post 2020
Patients	108	22	86
Median age (years)	30	35	28
Mean age+SD	31	33.2+13.14	30.12+8.39
Sex: male	75	12 (54.5%)	64 (74.4%)
Sex: female	33	10 (45.4%)	22 (25.6%)

We describe some of the main characteristics of these patients including admission status, requirement for surgery, ICU, and ventilation, as seen in Table 2. Of the 106 patients admitted to the hospital, pre 2020 included 90.1% of the patients whereas post 2020 included 100%. The pre-2020 group admitted to ICU included 4.5% vs 19.8% in the post-2020 group. 0% of the pre-2020 group required ventilation vs 3.5% in the post-2020 group. Lastly, 81.8% of the pre-2020 group required surgical intervention vs 96.5% of the post-2020 group. 

**Table 2:** Variables of injury severity ICU: intensive care unit

	All	Pre 2020	Post 2020	p-value
Total patients	108	22	86	
Admitted to hospital	106 (98.1%)	20 (90.1%)	86 (100.0%)	0.16
Required surgery	101(93.5%)	18 (81.8%)	83 (96.5%)	0.10
Required ICU	18 (16.7%)	1 (4.5%)	17 (19.8%)	-
Required ventilation	3 (2.8%)	0 (0.0%)	3 (3.5%)	-

We compared the ISS between the two groups, shown in Table 3. The median ISS for the pre-2020 group is 9+3.47 vs 15+6.66 for the post-2020 group. The p-value was found to be 0.21, deeming this result as not statistically significant. 

**Table 3:** ISS SD: standard deviation; ISS: injury severity score

	Pre 2020	Post 2020
Mean (+SD)	8.5 (+3.47)	9.81 (+6.66)
Median	9	15
p-value	0.21	

Lastly, we compared the days patients spent in the hospital, in the ICU, and on the ventilator, shown in Table 4, Table 5, and Table 6, respectively. The results show that in the pre-2020 group, the average days spent in the hospital was 9.32 days with a standard deviation (SD) of 9.56 days. The post-2020 group had an average of 10.83 days with an SD of 10.63. The pre-2020 group median was five days vs 6.5 days in the post-2020 group. Statistical analysis showed a p-value of 0.52, which is not statistically significant. For the days spent in the ICU, the pre-2020 group had a total of three days vs 100 days in the post-2020 group. The mean in the pre-2020 group was 0.14 days with an SD of 0.64 days vs 1.16 days with an SD of 3.80 in the post-2020 group. Statistical analysis showed a p-value of 0.02, allowing us to conclude the results to be statistically significant. The pre-2020 group did not have any patients that needed the ventilator, in contrast to the post-2020 group that had three patients on the ventilator for a total of 29 days. The mean in the post-2020 group was 0.34 days with an SD of 2.04 days. Statistical analysis showed the p-value to be 0.13, which is statistically insignificant. 

**Table 4:** Total LOS SD: standard deviation; LOS: length of stay in the hospital

	Pre 2020	Post 2020
Mean (+SD)	9.32 (+9.56)	10.83 (+10.63)
Median	5	6.5
p-value	0.52	

**Table 5:** Days spent in the ICU SD: standard deviation; ICU: intensive care unit

	Pre 2020	Post 2020
Total patients	1	17
Cumulative days	3	100
Mean (+SD)	0.14 (+0.64)	1.16 (+3.80)
P-value	0.02	

**Table 6:** Days on ventilator SD: standard deviation

	Pre 2020	Post 2020
Total days	0	29
Mean (+SD)	0	0.34 (+2.04)
p-value	0.13	

Although the analysis shows some statistically insignificant values, it is worth noting that the average and median days spent in the hospital, days spent in the ICU, and days spent on ventilator increased in the post-2020 group. This data is in alignment with the trends we noted in ISS, hospital admissions, and surgery, all of which increased in the post-2020 group. 

## Discussion

In this paper, we present a retrospective study to focus on the differences in the severity of injuries sustained from border wall falls before and after wall height increase. The mean ISS was greater in the post-2020 group as was the number of hospital admissions. The ICU admissions was found to be statistically significant with a p-value of 0.02, highlighting that the increased severity of injuries in the post-2020 group was associated with the increased wall height. No statistically significant differences were observed in the number of admissions, surgery, LOS, or ventilation days. However, the lack of statistical significance does not diminish the impact of this data. So while our data does not definitively conclude that patients who fall from the border wall are more severely injured since the height of the wall increased, it does show that there is an increase in volume which can direct us towards further understanding the implications of the raised border wall height [7,8].

Studies from the University of California San Diego (UCSD) Medical Center also hypothesized that the increase in border wall height is associated with increased injuries. Other previous research showed that there was an increase in incidence of injuries when the border wall height was extended from 10 feet to 15 feet from 2000 to 2007 [1,3]. Our study agrees with the study from UCSD that the number of injuries rose up dramatically after the increase in border wall height.

Several limitations are acknowledged in this report. Our data included patients admitted to just one level 1 trauma center in the region, while there are two other centers that serve patients who are also admitted from border wall falls. The small sample size limits our data by excluding those who were admitted to trauma centers in the San Diego area. Our data also does not include either patients who never sought out medical attention after sustaining a fall or patients whose injuries were so severe and died on the scene of the fall.

Additionally, although the wall has been reported to rise, there are areas along the border that have not been completed so it can be difficult to take into consideration those patients who crossed the border but didn't have to cross over the wall. We must also discern that the rise in volume may also be due to the fact that more people are migrating at a faster rate than ever before, which can also be contributing to the rise in the number of injuries.

Another important factor to consider is that the post-2020 numbers of patients may even be underreported due to the ongoing pandemic that burdened every single hospital in the nation and filled every emergency room to its capacity. This could have likely led to many patients who needed care that the hospitals could not provide [5,6].

## Conclusions

In the present report, we review the recent literature reporting the association between increased border wall height and increase in injury severity, characterized by hospital admissions, LOS, ICU, surgery, and ventilation needs. We demonstrate that hospital admissions from border wall falls increase by 12.5-fold. This new trend that is supported by other studies shows that such an increase is not only a humanitarian crisis at the border but also a medical one. Thousands of migrants cross the US-Mexican border each year hoping to find a safe haven in the United States. As one of the busiest international land borders, migrants at San Diego, California, and Tijuana, Mexico, are at increased risk for major injuries that have lifelong consequences. It would be wise to reconsider the purpose of the border wall extension and perhaps consider safer ways to implement immigration control.
